# Differences in Advanced Magnetic Resonance Imaging in MOG-IgG and AQP4-IgG Seropositive Neuromyelitis Optica Spectrum Disorders: A Comparative Study

**DOI:** 10.3389/fneur.2020.499910

**Published:** 2020-09-30

**Authors:** Felix A. Schmidt, Claudia Chien, Joseph Kuchling, Judith Bellmann-Strobl, Klemens Ruprecht, Nadja Siebert, Susanna Asseyer, Sven Jarius, Alexander U. Brandt, Michael Scheel, Friedemann Paul

**Affiliations:** ^1^NeuroCure Clinical Research Center, Charité – Universitätsmedizin Berlin, Corporate Member of Freie Universität Berlin, Humboldt-Universität zu Berlin, and Berlin Institute of Health, Berlin, Germany; ^2^Department of Neurology, Charité – Universitätsmedizin Berlin, Corporate Member of Freie Universität Berlin, Humboldt-Universität zu Berlin, and Berlin Institute of Health, Berlin, Germany; ^3^Clinical and Experimental Multiple Sclerosis Research Center, Department of Neurology, Charité – Universitätsmedizin Berlin, Berlin, Germany; ^4^Berlin Institute of Health, Berlin, Germany; ^5^Experimental and Clinical Research Center, Max Delbrueck Center for Molecular Medicine and Charité – Universitätsmedizin Berlin, Corporate Member of Freie Universität Berlin, Humboldt-Universität zu Berlin, and Berlin Institute of Health, Berlin, Germany; ^6^Division of Molecular Neuroimmunology, Department of Neurology, University Hospital Heidelberg, Heidelberg, Germany; ^7^Department of Neurology, University of California, Irvine, Irvine, CA, United States; ^8^Department of Neuroradiology, Charité – Universitätsmedizin Berlin, Berlin, Germany

**Keywords:** NMOSD, AQP4, MOG, MRI, DTI, advanced imaging

## Abstract

**Aims:** To explore differences in advanced brain magnetic resonance imaging (MRI) characteristics between myelin oligodendrocyte (MOG) immunoglobulin (IgG) and aquaporin-4 (AQP4) IgG seropositive (+) neuromyelitis optica spectrum disorders (NMOSD).

**Methods:** 33 AQP4-IgG and 18 MOG-IgG seropositive NMOSD patients and 61 healthy control (HC) subjects were included. All 112 participants were scanned with the same standardized MRI-protocol on a 3-Tesla MRI-scanner. Brain volume and diffusion tensor imaging (DTI) parameters were assessed.

**Results:** MOG-IgG+ patients showed reduced parallel diffusivity within white matter tracts compared to HC whereas AQP4-IgG+ showed no significant brain parenchymal damage in DTI analysis. AQP4-IgG+ patients showed reduced whole brain volumes and reduced volumes of several deep gray matter structures compared to HC whereas MOG-IgG+ patients did not show reduced brain or deep gray matter volumes compared to HC.

**Conclusions:** Microstructural brain parenchymal damage in MOG-IgG+ patients was more pronounced than in AQP4-IgG+ patients, compared with HC, whereas normalized brain volume reduction was more severe in AQP4-IgG+ patients. Longitudinal imaging studies are warranted to further investigate this trend in NMOSD. Our results suggest that MOG-IgG+ and AQP4-IgG+ NMOSD patients differ in cerebral MRI characteristics. Advanced MRI analysis did not help to differentiate between MOG-IgG+ and AQP4-IgG+ patients in our study.

## Introduction

Neuromyelitis optica spectrum disorders (NMOSD) are autoimmune diseases that present with longitudinally extensive transverse myelitis (LETM) and or optic neuritis (ON). They can also present with area postrema, diencephalic, cerebral or acute brainstem syndromes (1–6). The proof of serum autoantibodies directed against aquaporin-4 (AQP4-IgG) in around 80% of cases established NMOSD as a distinct disease from multiple sclerosis (MS) (7–10). In a subgroup of AQP4-IgG negative NMOSD patients serum antibodies targeting the myelin oligodendrocyte glycoprotein (MOG-IgG) were detected (11–16). MOG-IgG+ patients with clinical and neuroimaging characteristics of NMOSD are currently discussed as a different disease entity (17–23). Cortical encephalitis and seizures or cranial nerve involvement have also been reported in MOG-IgG+ patients (24–27).

While brain atrophy and microstructural tissue damage occur in MS from earliest disease stages (28–30), non-conventional MRI studies have shown conflicting results in NMOSD (31–35), and advanced imaging, such as diffusion tensor imaging (DTI) analysis, have not been reported from MOG-IgG+ patients.

Thus, the goal of our study was to further investigate possible MRI differences between MOG-IgG+ and AQP4-IgG+ mediated pathology in NMOSD patients and to compare these effects to a group of HCs.

## Materials and Methods

### Study Participants and Controls

Data for this cross-sectional study were extracted from an ongoing longitudinal observational study following patients with NMOSD and HCs performed at the NeuroCure Clinical Research Center, Charité Universitaetsmedizin Berlin.

Inclusion criteria were diagnosis of NMOSD according to the international consensus diagnostic criteria for NMOSD 2015 (36) or positive proof of MOG-IgG serum antibodies in a live cell-based assay and an associated demyelinating CNS disease with a clinical phenotype equivalent to NMOSD diagnosis criteria in patients over 18 years (17). In this regard, we treated MOG-IgG positivity as equivalent to positive AQP4-IgG for fulfilling the diagnostic criteria. All MOG-IgG+ patients met the currently proposed criteria for MOG encephalomyelitis (26). Exclusion criteria were a relapse within 3 months prior to MRI examination. Patients from the outpatient clinics of the Experimental and Clinical Research Center, NeuroCure Clinical Research Center and from the Department of Neurology, Charité – Universitätsmedizin Berlin were screened for eligibility.

### Ethics Statement

The study was approved by the Charité-Universitätsmedizin Berlin ethics committee (EA1/041/14) and was conducted in accordance to the Declaration of Helsinki in its currently applicable version and applicable German laws. All participants gave written informed consent to participate in the study.

## Clinical Data

### Clinical Assessment

The expanded disability status scale (EDSS) and further clinical characteristics were assessed on the day the MRI scan was performed as part of a study protocol. All patients included were enrolled into a prospective observational cohort study of NMOSD at NeuroCure Clinical Research Center and received a MRI scan annually as part of this cohort study. All patients were examined with the same standardized MRI protocol at the same MRI scanner for study purposes. From the majority of patients no MRI at disease onset was available because most of the included patients were referred from other hospitals and the department of neurology - Charité – Universitätsmedizin Berlin.

### MRI Acquisition Protocol

The multimodal brain MRIs all were acquired from a 3 T MRI (MAGNETOM Trio Tim, Siemens, Erlangen, Germany) and included a 3-dimensional T1-weighted magnetization prepared rapid acquisition gradient echo (MPRAGE) sequence (1 × 1 × 1 mm resolution, TR = 1,900 ms, TE = 3.03 ms), a 3-dimensional fluid attenuated inversion recovery (3D FLAIR) sequence (1 × 1 × 1 mm resolution, TR = 6,000 ms, TE = 388 ms) and a single-shot echo planar imaging DTI sequence (TR/TE = 7,500/86 ms; FOV = 240 × 240 mm; matrix 96 × 96, 61 slices no gap, slice thickness 2.3 mm, 64 non-collinear directions, b-value = 1,000 s/mm^2^).

### MRI-Data Post-processing

#### Brain and Deep Gray Matter Volumes

Gray and white matter volumes were calculated from MPRAGE scans, after lesion filling, using FSL SIENAX for normalized whole brain gray matter and white matter and with FSL FIRST for deep gray matter volumes (37–40).

## DTI Parameters

FSL DTIFIT (41) was used for DTI data processing, which included brain extraction and correction for eddy current distortions. Fractional anisotropy (FA) and parallel diffusivity were calculated by fitting a tensor model to the diffusion data using the tools from FSL. All data were then further processed with the tract based spatial statistics (TBSS) (42, 43).

### Statistical Analysis

Statistical analyses was were performed with R version 3.3.0 using the packages geepack and ggplot2 (44). Differences in demographics and clinical characteristics between patients and HC were tested using Welch's two-sample *t*-test. Differences in gender, clinical phenotype and immunosuppressive therapy were tested with the Fisher Exact Test. Group differences (brain volume and DTI measures) were analyzed using a linear regression model, including age, female sex and disease duration as a covariate. A *p*-value of <0.05 was considered significant. Due to the exploratory nature of the study, no correction for multiple comparisons was made.

## Results

From the 51 included patients, 33 had a diagnosis of AQP4-IgG seropositive NMOSD according to the 2015 IPND diagnostic criteria (36). From the 18 patients with positive MOG-IgG serology, five patients fulfilled the criteria for AQP4-IgG seronegative NMOSD. Another 13 patients were tested positive for serum MOG-IgG and had recurrent ON or at least one episode of LETM, but did not formally fulfill the 2015 IPND criteria for NMOSD. Demographics and clinical characteristics of AQP4-IG+ and MOG-IgG+ patients are summarized in Table 1. AQP4-IgG+ and MOG-IgG+ patients showed differences in sex and EDSS score, with no significant difference in age, disease duration, annualized relapse rate and further clinical characteristics (see Table 1).

**Table 1:** Demographics and clinical characteristics of AQP4-IgG+ and MOG-IgG+ patients.

**Parameter**	**AQP4 (*n* = 33)**	**MOG (*n* = 18)**	***p*-value**
Sex (f/m)	30/3	11/7	**0.02**
Age in years	50.3 ± 13.6 (24–72)	44.3 ± 12.2 (22–59)	0.11
EDSS	4 (0–7)	2 (1–6)	**0.01**
Annualized Relapse Rate	0.8 ± 1.2	1.3 ± 1.2	0.28
Total number of relapses	3.6 ± 2.1	3.7 ± 2.5	0.89
Disease Duration in years	8 ± 7	8 ± 11	1.0
Immunosuppressive Therapy	28 (85%)	14 (78%)	0.70
**Clinical Phenotype**			
LETM + bilateral ON	14 (42%)	5 (28%)	0.37
Bilateral ON	3 (9%)	4 (22%)	0.23
LETM only	12 (36%)	3 (17%)	0.20

*F, female; M, Male; EDSS, Expanded Disability Status Scale; LETM, longitudinal extensive transverse myelitis; ON, optic neuritis. EDSS is presented as median (range). Age, annual relapse rate, total number of previous attacks and disease duration are presented as mean ± standard deviation. Clinical phenotype and immunosuppressive therapy are presented as total numbers and in %*.

### Brain Volume Analysis

**Figure 1:**
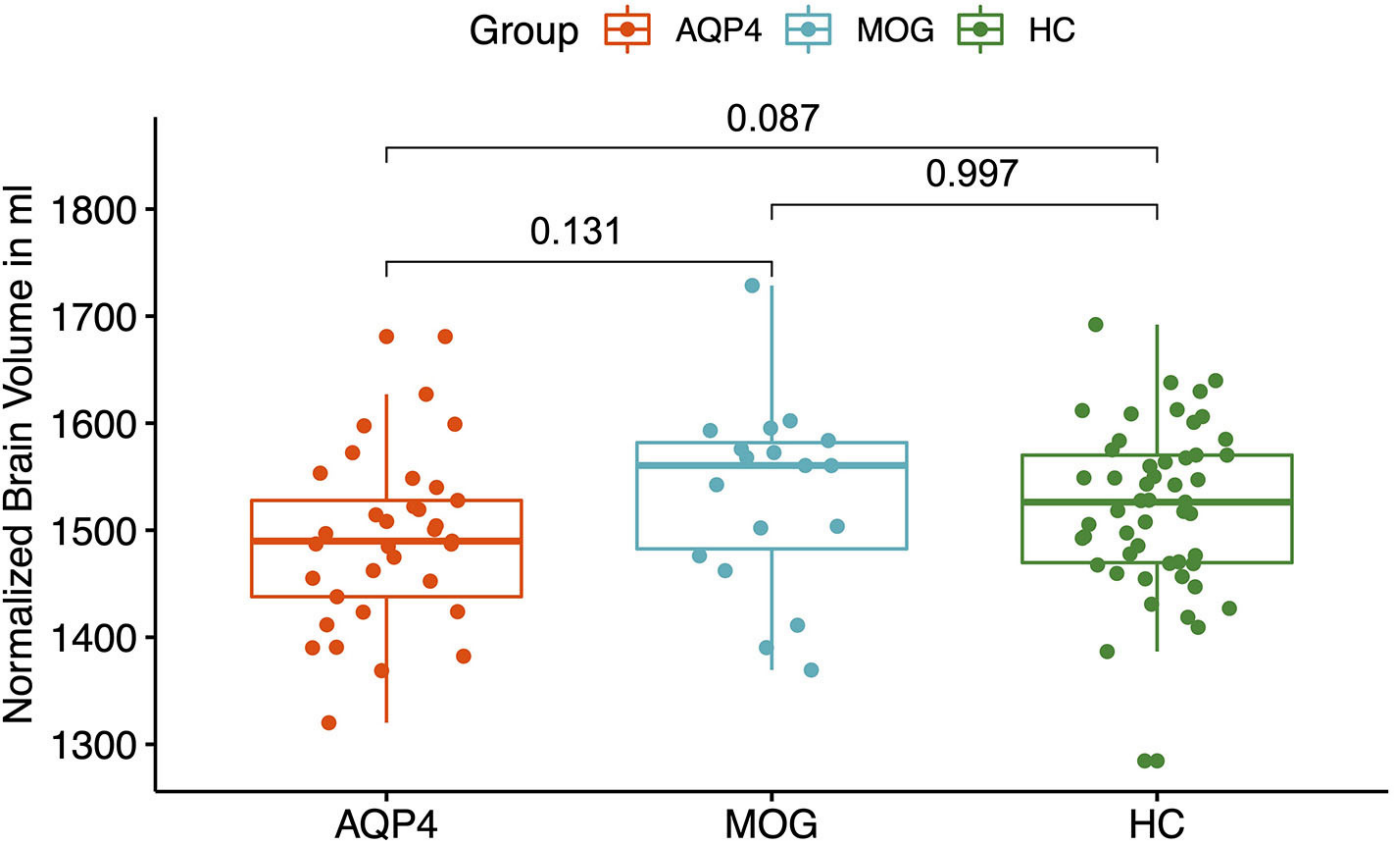
Normalized Brain Volumes. Y-axis: normalized brain volume in ml. X-axis: patient groups and healthy controls. AQP4, Aquaporin-4-IgG+ patients; HC, healthy control; MOG, Myelin oligodendrocyte glycoprotein IgG+ patients.

AQP4-IgG+ patients showed a trend for reduced normalized whole brain volumes compared to HC (*p* = 0.087, see Figure 1). There was no difference between AQP4-IgG+ patients, MOG-IgG+ patients and HC normalized gray matter volumes and white matter volumes.

### Brain Volume Analysis of Deep Gray Matter Structures

**Figure 2:**
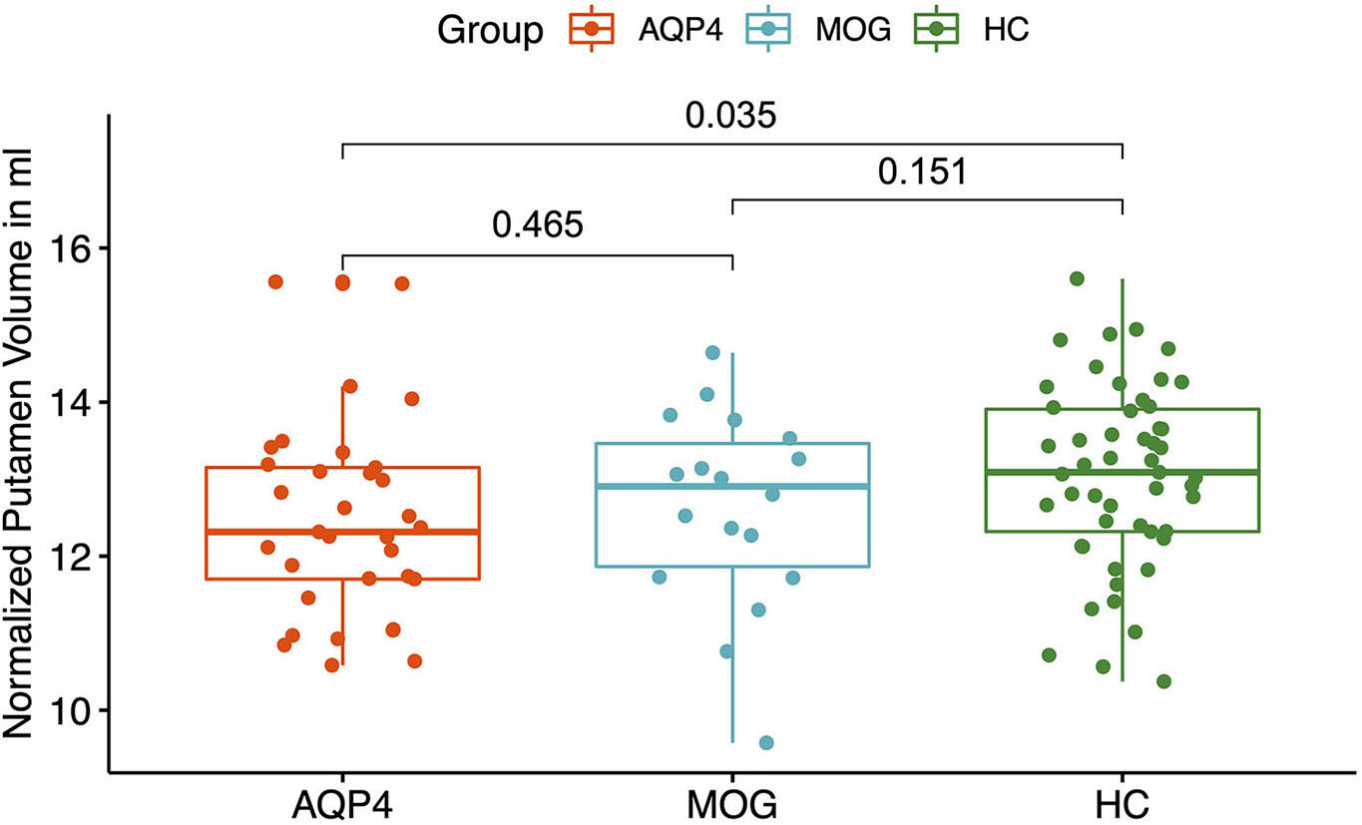
Y-axis: Normalized putamen volume in ml. X-axis: patient groups and healthy controls. AQP4, Aquaporin-4-IgG+ patients; HC, healthy control; MOG, Myelin oligodendrocyte glycoprotein IgG+ patients.

**Figure 3:**
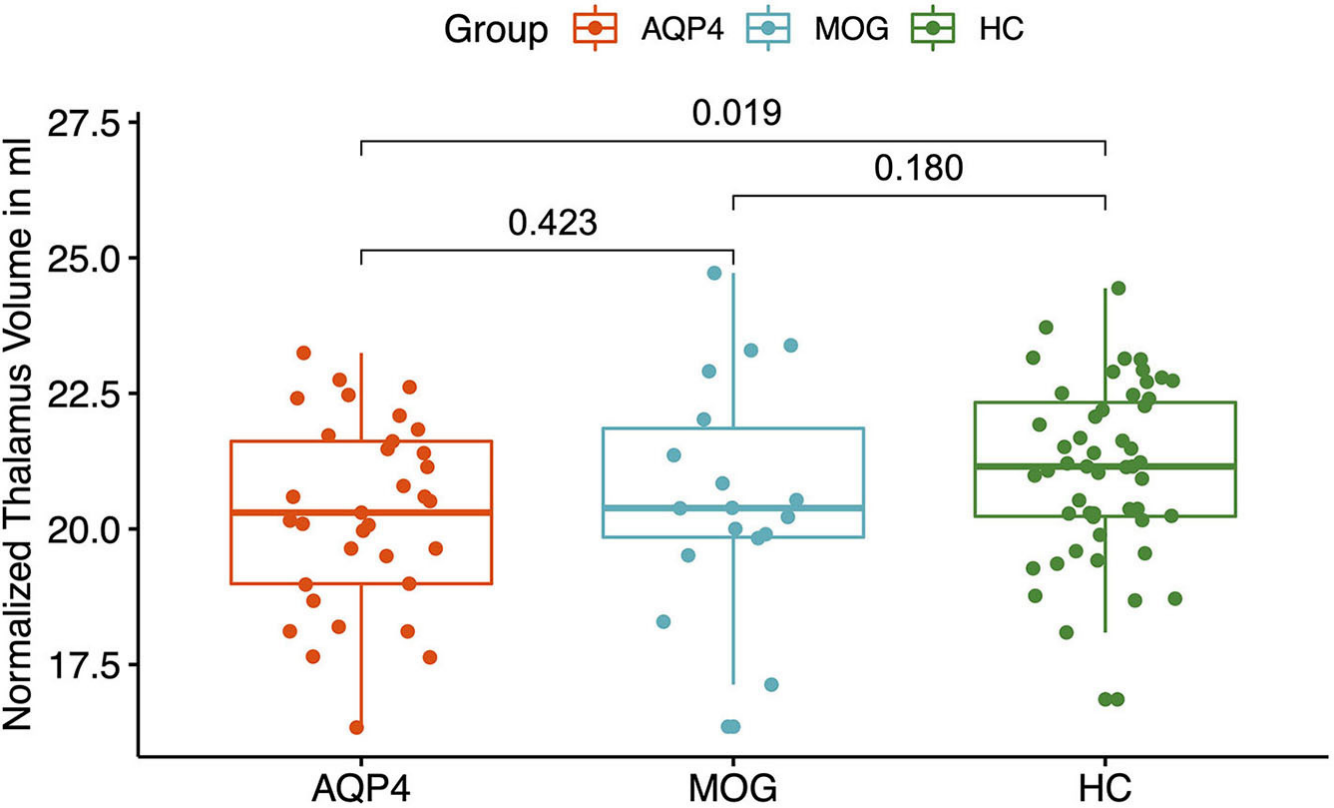
Y-axis: Normalized thalamus volume in ml. X-axis: patient groups and healthy controls. AQP4, Aquaporin-4-IgG+ patients; HC, healthy control; MOG, Myelin oligodendrocyte glycoprotein IgG+ patients.

**Figure 4:**
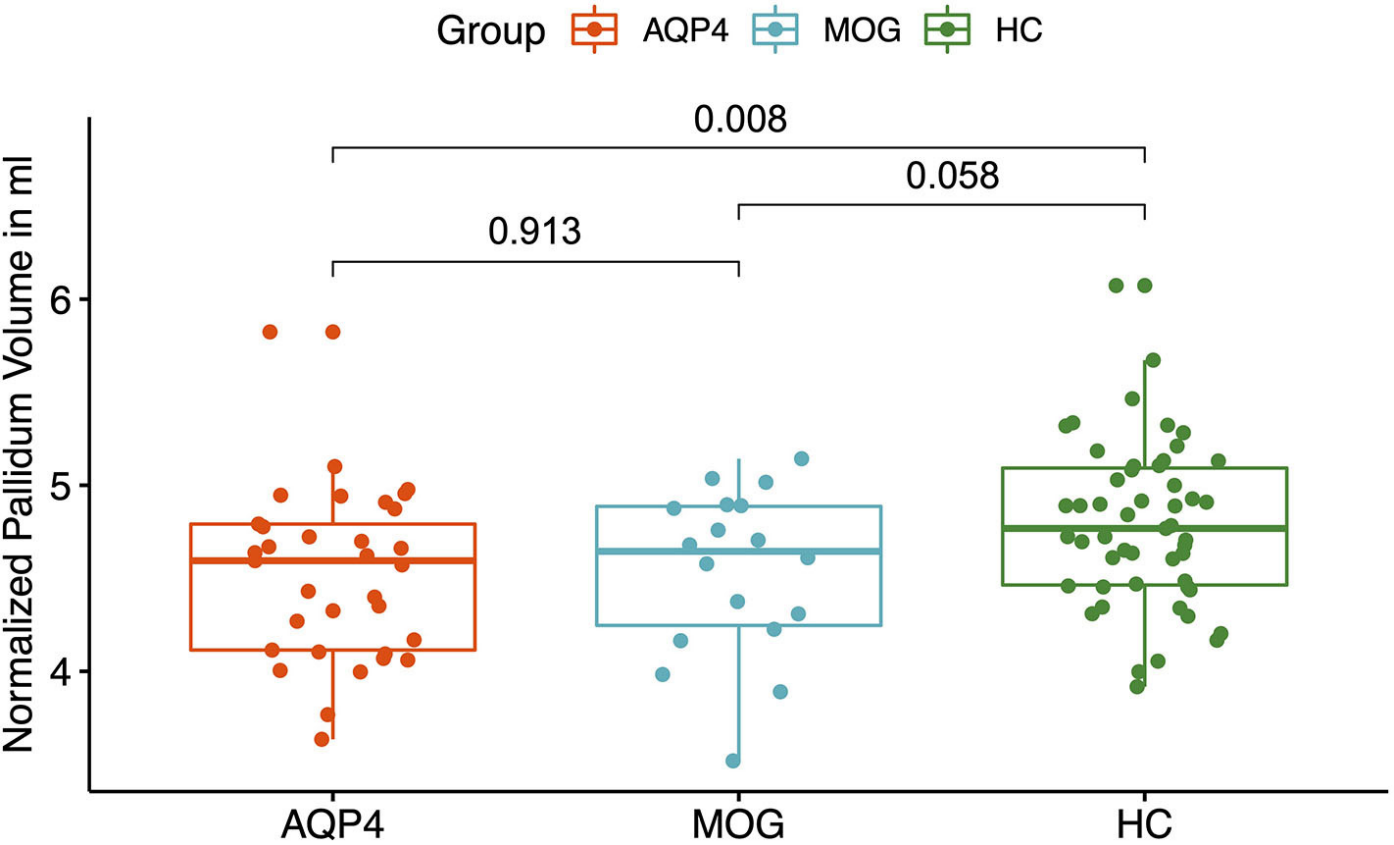
Y-Axis: Normalized pallidum volume in ml. X-axis: patient groups and healthy controls. AQP4, Aquaporin-4-IgG+ patients; HC, healthy control; MOG, Myelin oligodendrocyte glycoprotein IgG+ patients.

MOG-IgG+ patients did not show any volume reduction in deep gray matter structures compared to HC. A significant volume reduction for AQP4-IgG+ patients compared to HC was found in the putamen (*p* = 0.035, see Figure 2), the thalamus (*p* = 0.019, see Figure 3) and the pallidum (*p* = 0.008, see Figure 4).

### Diffusion Tensor Imaging

**Figure 5:**
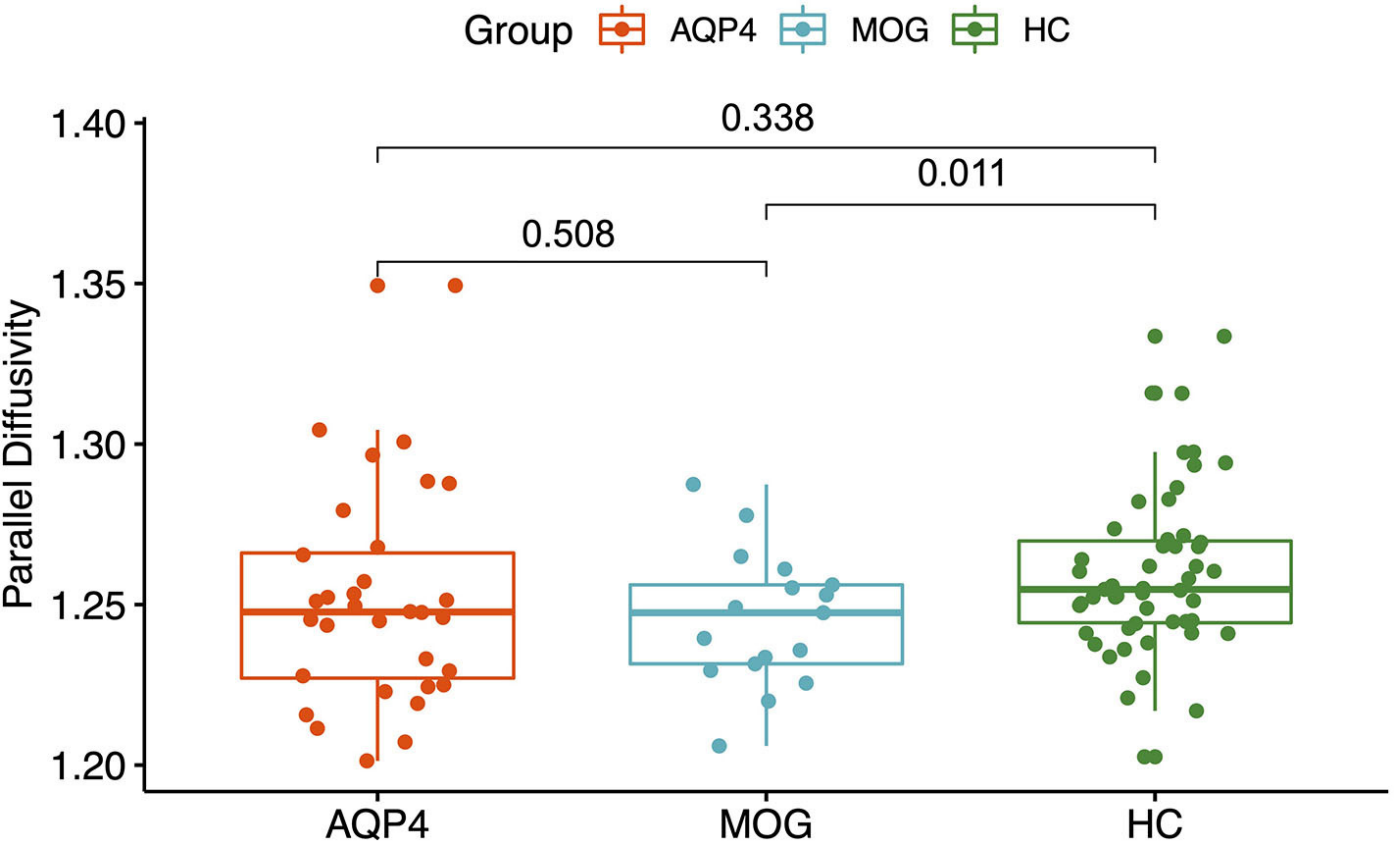
Y-axis: Parallel diffusivity. X-axis: patient groups and healthy controls. AQP4, Aquaporin-4-IgG+ patients; HC, healthy control; MOG, Myelin oligodendrocyte glycoprotein IgG+ patients.

MOG-IgG+ patients showed reduced parallel diffusivity compared to HC while AQP4-IgG+ patients showed no difference in parallel diffusivity compared to HC (see Figure 5). No difference was found between AQP4-IgG+ and MOG-IgG+ patients or HC in fractional anisotropy (FA), mean diffusivity, or radial diffusivity measures.

## Discussion

How intracranial volume and brain parenchymal damage differ between MOG-IgG+ and AQP4-IgG+ patients has not been investigated in detail before.

MOG-IgG seropositive patients with clinical and neuroimaging features of NMOSD are currently under discussion as being a distinct disease entity (45–51). There are few studies investigating MRI findings of MOG-IgG+ patients, likely due to its recent discovery and rare prevalence (18, 28, 46–48).

This is the first study that compares advanced MRI analysis of a larger cohort of MOG-IgG+ patients against AQP4-IgG+ patients along with HC. Other studies demonstrated that MRI brain lesion distribution criteria may help to distinguish MOG-IgG+ and AQP4-IgG+ patients from multiple sclerosis patients (28, 32, 45, 47, 48, 52). However, these studies did not analyze differences in brain volume and brain parenchymal damage.

Interestingly, in our brain volume analyses and DTI analysis no significant differences were found between MOG-IgG+ and AQP4-IgG+ patients, although MOG-IgG+ patients showed a reduced parallel diffusivity compared to HC. Our findings of reduced parallel diffusivity in white matter tracts of MOG-IgG+ patients compared to HC are complemented by previous studies investigating C57BL/6 mice with MOG-induced experimental autoimmune encephalomyelitis (EAE) that showed significant parallel diffusivity reduction within optic nerves (53) and within the spinal chord (54) with significant associations to EAE clinical scores and greater amounts of axonal damage as confirmed by quantitative staining. Decreased parallel diffusivity within the optic nerves of human patients after acute optic neuritis (55, 56) further corroborated the notion that decreased parallel diffusivity may be associated with various mechanisms of axonal damage, such as Wallerian degeneration, and diffuse axonal injury (57) that might have similarly occurred within the white matter of our MOG-IgG+ patients. Axonal loss and atrophy might lead to a bulk reduction of the intra-axonal volume and associated anisotropic diffusion profile (58). However, the exact biophysical mechanisms of reduction in parallel diffusivity still remain uncertain and further *in-* and *ex-vivo* studies are highly warranted to elucidate the exact associations between parallel diffusivity alterations and potentially corresponding axonal damage in patients with MOG-IgG+ encephalomyelitis.

AQP4-IgG+ patients showed a trend for a reduced normalized whole brain volume and a reduced volume of the putamen, pallidum and thalamus compared to HC. In a previous study, a smaller number of AQP4-IgG+ patients from our NMOSD cohort was analyzed and no significant brain volume reduction was found compared to HC with no brain parenchymal damage (34, 59). The differences could be explained by a statistical power problem, since the AQP4-IgG+ patient cohort was smaller.

Several measures were taken to establish a homogeneous study cohort to permit a comparison of these heterogeneous autoimmune disorders. Firstly, only antibody seropositive patients were included. Also, each patient was examined with the same standardized MRI protocol at the same MRI scanner. MRI data was evaluated by the same experienced neuroradiologist blinded to the patients' diagnosis. Further, the current study was restricted to mainly Caucasian patients with NMOSD given that ethnicity likely interacts with NMOSD pathogenicity with regard to possible genetic influence (i.e., Asian opticospinal MS) (60). Finally, AQP4-IgG+ and MOG-IgG+ patients were close in age where there was no significant difference in the means, thus reducing chances of finding differences in brain volume based on age.

A limitation of our study, and most of the previous imaging NMOSD studies, is the limited sample size due to the prevalence of this rare disease, especially in Caucasians (61). One possible solution to this fact, in a rare disease like MOG-IgG+ NMOSD, would be to compile available data in an international study group and pool imaging data in a common database for analysis in multicenter studies. This would allow for the evaluation of brain damage in NMOSD patients with enhanced statistical power. Another limitation are differences in sex and EDSS score between MOG-IgG+ and AQP4-IgG+ patients in our study. Both variables might have an impact on the MRI results.

We conclude that MOG-IgG+ patients in our cohort have more structural brain parenchymal damage, as detected by DTI measures when compared to HC whereas AQP4-IgG+ patients showed reduced whole brain volumes and reduced volumes of several deep gray matter structures compared to HC. Volume sub-analyses of deep gray matter structures and DTI measurements did not help to differentiate between MOG-IgG+- and AQP4-IgG+ patients in our study.

Our results are in favor of an early immunosuppressive treatment of patients with MOG encephalomyelitis whose treatment approaches still are a matter of debate (62). Similar to the treatment strategies for AQP4-IgG+ NMOSD patients, we believe this suggestion is valid because of the increased brain parenchymal damage observed from diffusion tensor imaging measures of parallel diffusivity in MOG-IgG+ patients. Longitudinal imaging studies are warranted to further investigate this trend of increased brain parenchymal damage in MOG-IgG+ patients.

## Data Availability Statement

The datasets generated for this study are available on request to the corresponding author.

## Ethics Statement

The studies involving human participants were reviewed and approved by Charité-Universitätsmedizin Berlin ethics committee (EA1/041/14). The patients/participants provided their written informed consent to participate in this study.

## Author Contributions

Study concept and coordination was performed by FP, AB, and MS. Data acquisition and interpretation were performed by FS and MS. Initial draft was performed by FS. Study supervision by FP, AB, and MS. Critical revision of the manuscript for important intellectual content was performed by FP, MS, AB, CC, KR, JK, NS, JB-S, SA, and SJ. All authors contributed to the article and approved the submitted version.

## Conflict of Interest

FS served on the scientific advisory board of Novartis, received travel funding and/or speaker honoraria from Biogen Idec, Bayer Healthcare, Teva Pharmaceuticals and Sanofi-Aventis/Genzyme. JK received conference registration fees from Biogen and financial research support from Krankheitsbezogenes Kompetenznetzwerk Multiple Sklerose (KKNMS), not related to this work. JB-S received travel funding and speaking fees from Bayer Healthcare, Sanofi-Aventis/Genzyme, and Teva Pharmaceuticals. KR served on the scientific advisory board for Sanofi-Aventis/Genzyme, Novartis, and Roche; received travel funding and/or speaker honoraria from Bayer Healthcare, Biogen Idec, Merck Serono, Sanofi-Aventis/Genzyme, Teva Pharmaceuticals, Novartis, and Guthy Jackson Charitable Foundation; is an academic editor for PLoS ONE; receives publishing royalties from Elsevier; and received research support from Novartis and German Ministry of Education and Research. SA received travel funding from Celgene. AB served on the scientific advisory board for Biogen; received travel funding and/or speaker honoraria from Novartis and Biogen; has patents pending from method and system for optic nerve head shape quantification, perceptive visual computing based postural control analysis, multiple sclerosis biomarker, and perceptive sleep motion analysis; has consulted for Nexus and Motognosis; and received research support from Novartis Pharma, Biogen Idec, BMWi, BMBF, and Guthy Jackson Charitable Foundation. FP serves on the scientific advisory board for Novartis; received speaker honoraria and travel funding from Bayer, Novartis, Biogen Idec, Teva, Sanofi-Aventis/Genzyme, Merck Serono, Alexion, Chugai, MedImmune, and Shire; is an academic editor for PLoS ONE; is an associate editor for Neurology® Neuroimmunology & Neuroinflammation; consulted for SanofiGenzyme, Biogen Idec, MedImmune, Shire, and Alexion; and received research support from Bayer, Novartis, Biogen Idec, Teva, Sanofi-Aventis/Genzyme, Alexion, Merck Serono, German Research Council, Werth Stiftung of the City of Cologne, German Ministry of Education and Research, Arthur Arnstein Stiftung Berlin, EU FP7 Framework Program, Arthur Arnstein Foundation Berlin, Guthy Jackson Charitable Foundation, and National Multiple Sclerosis of the USA. MS holds a patent for manufacturing of phantoms for computed tomography imaging with 3D printing technology and received research support from Federal Ministry of Economics and Technology. The remaining authors declare that the research was conducted in the absence of any commercial or financial relationships that could be construed as a potential conflict of interest. The reviewer ML-P declared a past co-authorship with one of the authors FP to the handling editor.
